# Circadian Clock Component BMAL1 in the Paraventricular Nucleus Regulates Glucose Metabolism

**DOI:** 10.3390/nu13124487

**Published:** 2021-12-15

**Authors:** Masanori Nakata, Parmila Kumari, Rika Kita, Nanako Katsui, Yuriko Takeuchi, Tomoki Kawaguchi, Toshiya Yamazaki, Boyang Zhang, Shigeki Shimba, Toshihiko Yada

**Affiliations:** 1 Department of Physiology, Wakayama Medical University School of Medicine, Kimiidare 811-1, Wakayama 641-8509, Japan; d1766026@wakayama-med.ac.jp (R.K.); d1866028@wakayama-med.ac.jp (N.K.); d1866052@wakayama-med.ac.jp (Y.T.); d1866030@wakayama-med.ac.jp (T.K.); zhangby@wakayama-med.ac.jp (B.Z.); 2 Department of Biotechnology, University of Wroclaw, Plac Uniwersytecki 1, 50-137 Wroclaw, Poland; parmi.choudhary89@gmail.com; 3 Department of Health Sciences, Kansai University of Health Sciences, Wakaba 2-11-1, Kumatoricho, Sennan-gun, Osaka 590-0482, Japan; yamazaki@kansai.ac.jp; 4 Laboratory of Health Science, School of Pharmacy, Nihon University, 7-7-1 Narashinodai, Funabshi 274-8555, Japan; shimba.shigeki@nihon-y.ac.jp; 5 Center for Integrative Physiology, Kansai Electric Power Medical Research Institute, 1-5-6 Minatojimaminamimachi, Chuou-ku, Kobe 650-0047, Japan; toshihiko.yada@kepmri.org; 6 Division of Diabetes, Metabolism and Endocrinology, Kobe University Graduate School of Medicine, Kusunokicho 7-5-1, Chuou-ku, Kobe 650-0017, Japan

**Keywords:** paraventricular nucleus, circadian, BMAL1, vasopressin, insulin release, glucose metabolism

## Abstract

It is suggested that clock genes link the circadian rhythm to glucose and lipid metabolism. In this study, we explored the role of the clock gene *Bmal1* in the hypothalamic paraventricular nucleus (PVN) in glucose metabolism. The *Sim1*-*Cre*-mediated deletion of *Bmal1* markedly reduced insulin secretion, resulting in impaired glucose tolerance. The pancreatic islets’ responses to glucose, sulfonylureas (SUs) and arginine vasopressin (AVP) were well maintained. To specify the PVN neuron subpopulation targeted by Bmal1, the expression of neuropeptides was examined. In these knockout (KO) mice, the mRNA expression of *Avp* in the PVN was selectively decreased, and the plasma AVP concentration was also decreased. However, fasting suppressed *Avp* expression in both KO and Cre mice. These results demonstrate that PVN BMAL1 maintains *Avp* expression in the PVN and release to the circulation, possibly providing islet β-cells with more AVP. This action helps enhance insulin release and, consequently, glucose tolerance. In contrast, the circadian variation of *Avp* expression is regulated by feeding, but not by PVN BMAL1.

## 1. Introduction

Living organisms, including humans, have developed to adapt to the 24 h day–night cycle governing their daily activities. This is achieved by their own biological clock system, which controls daily rhythms of the endocrine system and metabolism. Glucose, a major fuel source for mammals, is strictly regulated to maintain normal blood glucose levels. The blood glucose levels exhibit a clear circadian rhythm in healthy human subjects [1,2]. Glucose metabolism follows the circadian rhythm, reflected by the circadian variation of glucose tolerance that peaks during the early light period, when feeding typically occurs, and declines during the dark period, when fasting typically occurs [3]. Metabolic disorders, including obesity and type 2 diabetes mellitus (T2DM), have reached pandemic levels in modern human societies. Irregular meals associated with working night shifts disturb the circadian rhythm, possibly impairing glucose tolerance [4]. In addition to the imbalance between total calorie intake and total energy expenditure, disruptions in biological rhythms increase the risk of developing obesity and T2DM.

The circadian rhythm is based on a complex program of clock gene expression [5]. In mammals, CLOCK and brain and muscle ARNT-like 1 (BMAL1) form a heterodimeric complex and transcriptionally act as positive regulators of circadian genes, cryptochromes (CRYs) and periods (PERs). When PER and CRY proteins reach critical levels, they repress the expression of CLOCK and BMAL1. Subsequently, the proteolytic degradation of PERs and CRYs de-represses the expression of CLOCK and BMAL1. This negative feedback regulation forms a self-sustainable cycle that repeats itself every 24 h.

The importance of the molecular clock in glucose homeostasis was revealed by pronounced metabolic phenotypes in *Clock* mutant mice [6]. These animals exhibited hyperphagia, obesity, hyperleptinemia, hyperlipidemia, hyperglycemia and hypo-insulinemia. For instance, whole-body knockout (KO) mice of *Bmal1* show fasting hypoglycemia, reduced plasma insulin levels and increased adiposity [7]. *Per2* mutant mice exhibit impairment in gluconeogenesis and glucocorticoid oscillations [8]. *Cry1* and *Cry2* double KO mice exhibit severely impaired glucose clearance despite normal responsiveness to insulin [9]. Abnormal glucose metabolism has been reported in circadian clock mutant mice.

The circadian rhythm of glucose metabolism is orchestrated by an interplay of central and peripheral clocks, with a master pacemaker located in the suprachiasmatic nucleus (SCN) [10]. Clocks are also present in peripheral tissues, such as the liver, adipose tissue and pancreatic islets. Under rhythmic light–dark conditions, the SCN is entrained to the external light cycle and coordinates the circadian rhythm of tissues, resulting in oscillating levels of various metabolic parameters. The SCN projects to the paraventricular nucleus (PVN) of the hypothalamus; the functional importance of this SCN–PVN connection in controlling plasma glucose concentrations was revealed by pharmacological treatment into the SCN and the PVN [11,12].

The neurons of the PVN express a vast array of neuropeptides that are critically involved in a number of homeostatic functions [13]. Neuronal populations within the parvocellular division express two trophic hormones, thyrotropin-releasing hormone (TRH) and corticotropin-releasing hormone (CRH). The magnocellular division synthesizes two peptides, oxytocin and arginine vasopressin (AVP). The PVN neurons also express other neuropeptides, including nucleobindin-2/nesfatin-1 (NUCB2/Nesf-1), which participates in the control of energy metabolism [14]. With regard to blood glucose homeostasis, the PVN’s unique ability to integrate afferent signals from the hypothalamus including the SCN and extra-hypothalamic areas such as the nucleus of the solitary tract (NTS) of the medulla positions it as a key player in the regulation of glucose metabolism. In particular, some neurons in the SCN project to the CRH neurons in the PVN, which dictates the circadian rhythm of circulating glucocorticoids [15]. These findings suggest that the autonomous clock in the SCN controls the molecular clock in the PVN. Furthermore, clock genes including BMAL1 are located not only in the SCN but also in the PVN. Therefore, BMAL1 potentially regulates the circadian rhythm of endocrine and metabolic systems. However, the functional relevance of the interaction between Bmal1 and the PVN neurons, including AVP, CRH, oxytocin and NUCB2/Nesf-1, in glucose metabolism remains unknown. To explore whether the circadian rhythm of PVN neurons regulates glucose metabolism, we generated PVN-specific Bmal1 null mice and studied glucose tolerance in these mice.

## 2. Materials and Methods

### 2.1. Animals

We generated PVN-specific Bmal1 knockout (KO) mice by mating Sim1-Cre-transgenic (Cre) mice (a generous gift from Dr. Joel K. Elmquist, University of Texas) with *Bmal1 ^flox/flox^* mice [16,17]. *Bmal1 ^flox/flox^* mice were crossed with *Sim1*-*Cre* mice for one generation to generate *Sim1*-*Cre: Bmal1 ^flox /−^* mice, followed by interbreeding of offspring to yield *Sim1*-*Cre*: *Bmal1 ^flox/flox^* (KO) mice, or *Sim1*-*Cre* mice littermate controls. All mice were genotyped by polymerase chain reaction (PCR) amplification of genomic DNA isolated from tail tips. Mice were maintained under a 12 h light/dark cycle (8:00 a.m. lights on) and given standard food CE-2 (Japan SLC, Tokyo, Japan) and water ad libitum. Male C57BL/6 mice (SLC, Hamamatsu, Japan) were maintained in the same conditions as for KO mice. All experimental protocols were approved by the Wakayama Medical University Animal Care and Use Committee.

### 2.2. Immunofluorescence Staining of BMAL1

Briefly, animals were perfused transcardially using phosphate buffer containing 4% paraformaldehyde and 0.2% picric acid. Brain samples were stained for BMAL1 following previous reported methods with slight modifications [18]. Briefly, rabbit anti-BMAL1 (Abcam, Cambridge, UK; 1:1000) was used as the primary antibody and Alexa fluor 488 goat anti-rabbit (Life Technologies, Carlsbad, CA, USA; 1:500) as the secondary antibody.

### 2.3. Glucose Tolerance and Insulin Tolerance Tests

For glucose tolerance tests, Cre mice and KO mice aged 12 weeks old were fasted for 4 h. Following administration of glucose (1 g per kg i.p.) at zeitgeber time (ZT5), glucose levels were measured in tail blood at the indicated time points by glucocard (Arkay, Kyoto, Japan). Plasma insulin content was determined by enzyme immunoassay using a kit (Shibayagi, Gunma, Japan). For insulin tolerance tests, mice were fasted for 4 h. Following administration of insulin (1 IU per kg i.p.) at ZT5, glucose levels were measured in tail blood at indicated time points.

### 2.4. Preparation of Islets and Measurements of Insulin Release

Islets of Langerhans were isolated from mice aged 12 weeks by collagenase digestion according to a previously reported method [19]. Groups of 10 islets were first incubated at 37 °C for 30 min in HKRB with 2.8 mmol/L glucose, followed by the test incubation for 30 min in HKRB containing 2.8 or 8.3 mmol/L glucose without or with test agents. Insulin content was determined by ELISA (Shbayagi, Shibukawa, Japan).

### 2.5. RT-PCR

All procedures were performed as previously reported [20]. Briefly, bilateral PVNs were immediately resected from hypothalamic slices from Cre mice and KO mice at ZT1 and ZT13. Total RNA was isolated by TRIzol (Invitrogen, Carlsbad, CA, USA) and cleaned up with RQ1-DNase (Promega, Madison, WI, USA). First-strand cDNA synthesis was completed using ReverTra Ace kit (TOYOBO, Osaka, Japan). Quantitative real-time PCR assay was performed using SYBR Premix Ex Taq II polymerase (Takara bio, Shiga, Japan) in Thermal Cycler Dice (Takara bio, Shiga, Japan), and data were analyzed by the ΔΔCT method of relative quantification. The expression levels of mRNA were normalized to the product glyceraldehyde-3-phosphate dehydrogenase (*Gapdh*). Primers are indicated in Table 1.

### 2.6. Measurement of Plasma AVP Concentration

At ZT1, blood was collected in tubes containing EDTA and aprotinin and separated by centrifugation for 10 min at 3000× *g*. Plasma concentrations of AVP (Enzo Life Science, Famingdale, NY, USA) were measured with an enzyme immunoassay method. 

### 2.7. Measurement of mRNA Expression in the PVN of Ad Libitum-Fed and Fasted Mice

For the study under fasting conditions, food deprivation was started at ZT1. After 24 h, mice were deeply anesthetized, and their brains were removed. Brain slices containing the entire PVN were prepared, and the entire PVN was excised from the left and right sides.

### 2.8. Measurement of Serum Corticosterone Concentration

At ZT1 and AT13, blood was collected in tubes. Serum concentrations of corticosterone (Assypro, St Charles, MO, USA) were measured with an enzyme immunoassay method.

### 2.9. Statistical Analysis

Data were presented as mean ± SEM. One-way ANOVA was applied. Post hoc multiple comparison was generated using the Bonferroni test. A value of *p* < 0.05 was considered significant.

## 3. Results

**Figure 1:**
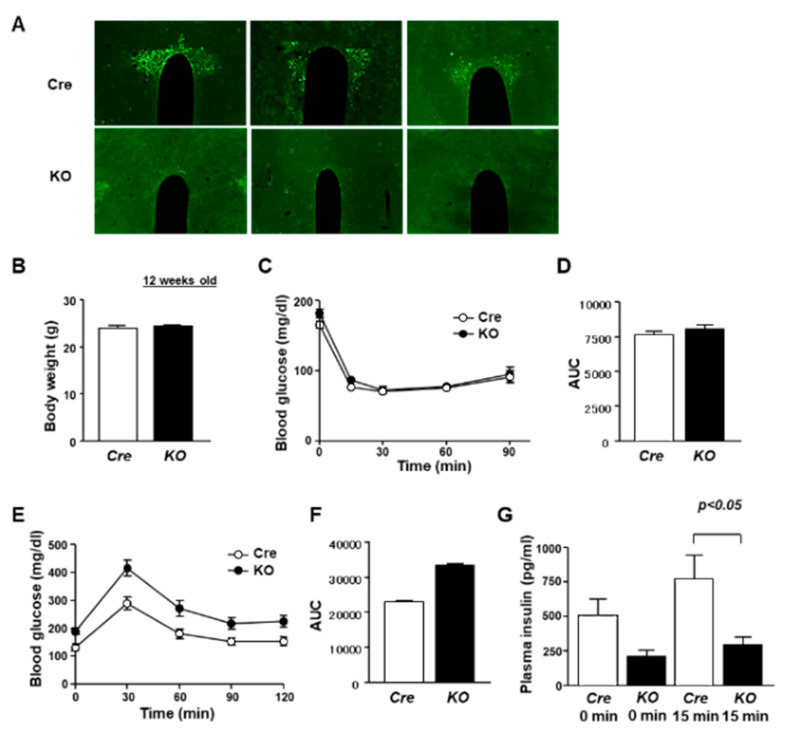
PVN-specific Bmal1 KO mice exhibited impairment of glucose intolerance. (**A**) Immunostaining for BMAL1 in PVN coronal sections (rostral–caudal) of Cre and KO mice. Upper: Cre mice, lower: KO mice. Metabolic phenotypes of KO mice. (**B**) Body weight in Cre mice and KO mice at 12 weeks of age (*n* = 6). (**C**) Blood glucose levels were measured in an ITT after intraperitoneal administration of insulin (1 IU per kg) to Cre mice (open symbol) and KO mice (filled symbol) at 12 weeks of age (each group, *n* = 6). (**D**) Area under the curve (AUC) of ITT (each group, *n* = 6). (**E**) Blood glucose during GTT after intraperitoneal administration of glucose (1 g per kg) to Cre mice (open symbol) and KO mice (filled symbol) at 12 weeks of age (each group, *n* = 6). (**F**) AUC of GTT (each group, *n* = 6). (**G**) Plasma insulin concentrations during GTT in Cre mice and KO mice (*n* = 6). Data are presented as mean ± SEM. Cre vs. KO determined by one-way ANOVA followed by the Bonferroni test.

The link of the molecular clock in the PVN to glucose metabolism is unclear. To address this issue, we generated PVN *Bmal1* KO mice by crossing Sim1-Cre mice and *Bmal1^ flox/flox^* mice. The *Sim1* gene is expressed predominantly in the PVN and supraoptic nucleus (SON) [21]. BMAL1 is expressed in the PVN of Cre mice (Figure 1A). In the KO mice, expression of immunofluorescence for BMAL1 was absent in the PVN and reduced in the SON (Figure 1A and Supplementary Figure S1). PVN-specific Bmal1 ablation affected neither body weight (Figure 1B) nor insulin sensitivity in the insulin tolerance test at 12 weeks of age (Figure 1C,D). Under fasting conditions, KO mice had markedly higher levels of plasma glucose than Cre mice (Time 0 in Figure 1E). In the glucose tolerance test (GTT), KO mice exhibited a markedly increased blood glucose level compared to Cre mice (Figure 1E,F). Moreover, insulin levels were significantly lower in KO mice compared to Cre mice at 0–15 min of the GTT (Figure 1G). These results indicate that PVN-specific Bmal1 ablation impaired insulin secretion, resulting in glucose intolerance.

**Figure 2:**
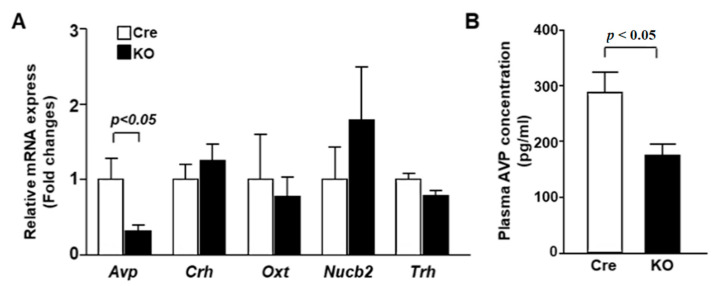
AVP mRNA expression in the PVN and plasma AVP concentration were decreased in KO mice. (**A**) The PVN of Cre mice and KO mice at 12 weeks of age was isolated at ZT1 for mRNA isolation (*n* = 6 for each group). The mRNA levels of *Avp*, *Crh*, *Oxt*, Nucb2 and *Trh* were measured by RT-PCR and expressed as values relative to the expression of Cre mice, which is arbitrarily defined as 1. (**B**) Plasma AVP concentrations at ZT1 in Cre and KO mice at 12 weeks of age (*n* = 6 for each group). Data are presented as mean ± SEM. Cre vs. KO determined by one-way ANOVA followed by the Bonferroni test.

To specify the altered PVN neuron subpopulation in KO mice, the expression of neuropeptides was examined. In the daytime (ZT1), mRNA expression of *Avp* was decreased in the PVN of KO mice, while *Crh*, *Nucb2*, *Oxt* and *Trh* mRNA expression was not altered (Figure 2A). Furthermore, the plasma concentration of AVP was markedly decreased in KO mice compared to Cre mice (Figure 2B). These results suggest that PVN-specific *Bmal1* ablation suppressed the expression of AVP in the PVN during the daytime, resulting in a lower plasma AVP concentration.

**Figure 3:**
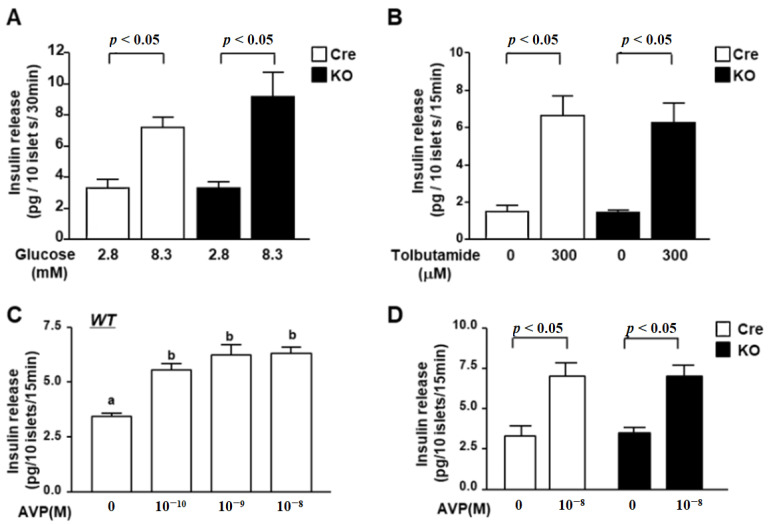
Glucose- and AVP-dependent increases in insulin release from islets of KO mice. (**A**) Insulin release from isolated islets under static incubation with 2.8 mM glucose and 8.3 mM glucose. High glucose (8.3 mM) significantly increased insulin secretion from isolated islets of both Cre mice (white bars) and KO mice (black bars), and the increased level was not significantly different between Cre and KO mice (*n* = 6 groups). (**B**) Insulin release from isolated mouse islets by tolbutamide (Tolb: 300 µM) at 2.8 mM glucose. There was no significant difference between Cre mice (white bars) and KO mice (black bars). (**C**) Insulin release from islets of wild C57B6 mice in the presence of 2.8 mM glucose with 10^−10^, 10^−9^ or 10^−8^ M AVP. Insulin release was stimulated by AVP in a concentration-dependent manner to reach a significant level with 10^−8^ M (*n* = 6). ^a,b^ Different letters indicate *p* < 0.05. (**D**) Insulin release from isolated islets under 2.8 mM glucose with 10^−8^ M AVP. AVP significantly increased insulin secretion from isolated islets of both Cre (white bars) and KO mice (black bars). Data are presented as mean ± SEM. Cre vs. KO, 2.8 G vs. 8.3 G, Vehicle vs. AVP, determined by one-way ANOVA followed by the Bonferroni test.

To elucidate the mechanism underlying the regulation of insulin secretion by PVN *Bmal1*, we investigated the insulin secretory function of pancreatic islets in KO and Cre mice. High glucose (8.3 mM)-stimulated insulin release from isolated islets from KO mice was similar to that from Cre mice (Figure 3A). Moreover, insulin release from islets of KO mice by stimulation with the SU tolbutamide (Tolb: 300 µM) was also not significantly different from that of Cre mice (Figure 3B).

It was reported that the receptor of AVP is expressed in β-cells and that AVP stimulates insulin release. Finally, we investigated the ability of insulin secretion in response to AVP in islets. Insulin release from islets of wild mice was stimulated by AVP in a concentration-dependent manner to reach a significant level with 10^−8^ M (Figure 3C). AVP at 10^−8^ M significantly increased insulin release from isolated islets of both Cre and KO mice to the same level (Figure 3D). These results indicate that KO mice maintain the ability of insulin release from islet β-cells at the same level as that of Cre mice.

**Figure 4:**
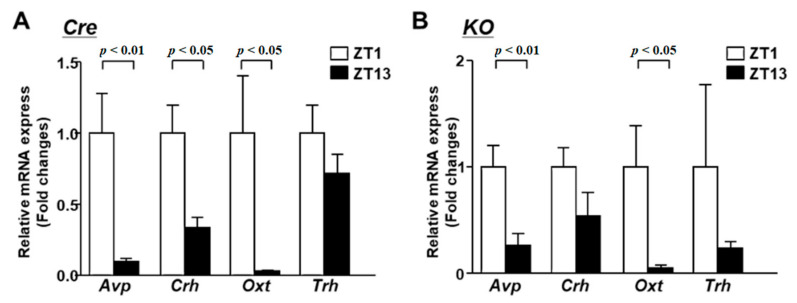
The mRNA expression in the PVN at light phase and dark phase. (**A**) The PVN of Cre mice at 12 weeks of age was isolated at 2 time points (ZT1 and ZT13) for mRNA isolation (*n* = 6 for each group). The mRNA levels of Avp, Crh, Oxt and Trh were measured by RT-PCR and expressed as values relative to the time point of ZT0, which is arbitrarily defined as 1. (**B**) Relative mRNA expression in the PVN at ZT1 and ZT13 of KO mice at 12 weeks of age (*n* = 6 for each group). Data are presented as mean ± SEM. ZT0 vs. ZT12 determined by one-way ANOVA with the Bonferroni multiple comparisons test.

Next, we analyzed the expression of endocrine-related neurotransmitters in the light and dark periods. In Cre mice, the expression of *Avp*, *Crh* and *Oxt* showed predominantly higher levels in the early period compared to in the dark period (Figure 4A). On the other hand, the expression of *Trh* did not change significantly between the light and the dark period. In KO mice, *Avp* and *Oxt* were highly expressed in the light period, as in Cre mice (Figure 4B). This result indicates that circadian variation in the expression of *Avp* and *Oxt* does not depend on the circadian clock or BMAL1 in the PVN.

However, the increased expression of *Crh* in the light period was not significant in KO mice. In addition, the rhythm of corticosterone secretion was also impaired in KO mice (Supplementary Figure S2). This result suggests that BMAL1 in the PVN regulates the circadian variation of corticosterone.

**Figure 5:**
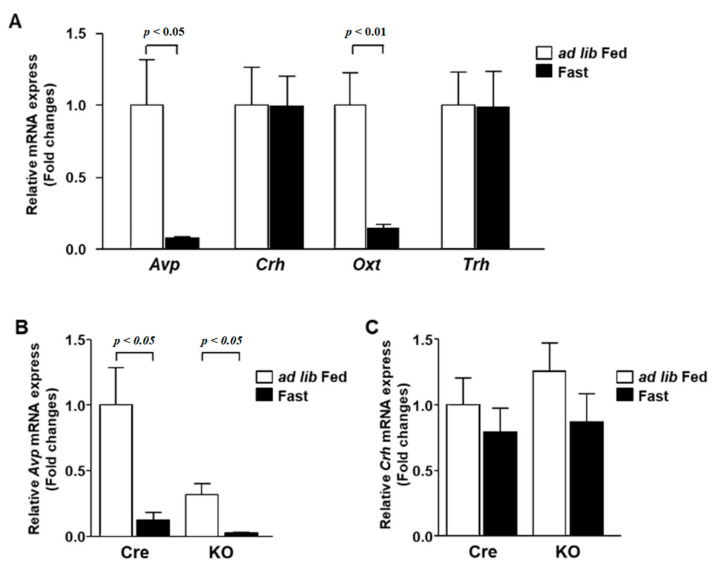
The mRNA expression in the PVN during fasting. (**A**) The PVN was isolated at ZT1 from 12-week-old C57BL/6 mice at ad lib feeding and after 24 h fasting conditions (*n* = 6 for each group). The mRNA levels of *Avp*, *Crh*, *Oxt* and *Trh* were measured by RT-PCR and expressed as values relative to the expression of ad lib-fed mice, which is arbitrarily defined as 1. (**B**,**C**) Relative mRNA expression of *Avp* (**B**) and *Crh* (**C**) in the PVN of Cre mice and KO mice at ad lib feeding and after 24 h fasting conditions (*n* = 6 for each group). Gene expression levels are represented as values relative to the expression of ad lib-fed Cre mice, which is arbitrarily defined as 1. Data are presented as mean ± SEM. Ad lib fed vs. fasting determined by one-way ANOVA with the Bonferroni multiple comparisons test.

Feeding conditions affect the activity of neurons in the PVN as much as the circadian rhythm. Therefore, we analyzed the gene expression in wild-type mice after a 24-h feeding restriction. The expression of both *Avp* and *Oxt* decreased under fasting conditions (Figure 5A). The expression of *Avp* in KO mice was lower than that in Cre mice in the ad libitum condition, but it was reduced by fasting (Figure 5B). On the other hand, the expression of *Crh* was not altered by fasting in both Cre mice and KO mice (Figure 5C).

## 4. Discussion

In this study, we showed that the deletion of *Bmal1* in the PVN markedly reduced insulin secretion, resulting in impaired glucose tolerance. The AVP mRNA expression in the PVN and plasma AVP concentration also decreased in KO mice, which may have lowered the amount of AVP that acts on islet β-cells to promote insulin secretion. Thus, BMAL1 in the PVN plays an important role in the regulation of glucose metabolism via mechanisms including the maintenance of AVP expression in the PVN.

It has been reported that AVP promotes insulin secretion [22,23]. Moreover, the AVP receptor V1b is expressed in pancreatic β-cells, and AVP directly activates pancreatic β-cells [23,24,25,26]. Furthermore, AVP provokes insulin release from β-cells at both stimulatory high-glucose and non-stimulatory low-glucose concentrations [26]. For humans, it has been suggested that the basal secretion of insulin during the early nighttime is important for the suppression of gluconeogenesis in the liver. Therefore, even in diabetic patients, long-acting insulin is used for treatment. AVP is also considered to be involved in basal insulin secretion. In this study, both AVP and high glucose increased insulin release from islets in control Cre and KO mice to a similar extent. These results indicate that decreased insulin secretion is caused by a decreased plasma AVP concentration and its insulinotropic action, while β-cell function is not altered.

The global Bmal1 KO mice exhibited impaired glucose tolerance with reduced insulin secretion [7]. In addition, deletion of Bmal1 in pancreatic β-cells also reduced insulin secretion [27]. These reports indicate that the clock genes are implicated in maintenance of the insulin secretory machinery in β-cells. In this study, we showed that BMAL1 expression in the PVN is also important in regulating insulin secretion via AVP, and that AVP plays a role as a humoral factor to stimulate insulin secretion.

It is well known that the release of AVP takes place in a circadian pattern, with the highest level during the early nighttime and the lowest level in the afternoon in humans [28]. Conversely, in rodents, the AVP concentration increases in the light period [29]. The circadian rhythm of AVP release is considered to be associated with the circadian clocks of AVP neurons in the PVN. In the present study, the deletion of Bmal1 in PVN neurons did not influence the fluctuation in *Avp* and *Oxt*. The expression of these two neurotransmitters has been regulated by dietary conditions [30,31], and in this study, fluctuations in the expression of both *Avp* and *Oxt* were observed in KO mice. However, the range of *Avp* expression in KO mice was weakened. In the SCN, BMAL1 binds to E-box in the promoter of the *Avp* gene and positively regulates the expression of *Avp* [32,33]. In an analogous way, it is speculated that neuronal input from the SCN and feeding conditions complicatedly regulate the circadian fluctuation in *Avp* expression, and BMAL1 may regulate the gain of this fluctuation. The PVN *Bmal1* deletion decreased the expression of *Avp* and, consequently, the release of AVP.

AVP, an unstable peptide, is rapidly cleared from plasma and is largely attached to platelets in the circulation. Copeptin, a cleavage product of the C-terminal part of the AVP precursor, has a long half-life and is found at higher concentrations than AVP in plasma [34]. Plasma levels of copeptin, usually used instead of AVP, are elevated in various diseases and metabolic syndrome [34,35]. In particular, high plasma copeptin was reported to be associated with reduced insulin sensitivity [34]. These reports suggest that AVP secretion is enhanced in metabolic syndrome. In addition, it was suggested that undesirable environmental factors often impair the circadian rhythm, such as circadian release of AVP, leading to the progression of metabolic syndrome with dysregulation of insulin secretion. The present study revealed a key role of PVN Bmal1 in regulating *Avp* expression and release, and, consequently, insulin release.

The release of glucocorticoids exhibits the exemplary circadian variation. In the hypothalamic–pituitary–adrenal (HPA) axis, CRH neurons in the PVN promote the release of adrenocorticotropin hormone (ACTH), thereby stimulating glucocorticoid secretion. The CRH neurons strongly receive neurological inputs from the SCN, and circadian rhythms in CRH expression synchronize to the activity of VIP neurons in the SCN [36]. Therefore, it is speculated that the fluctuation in CRH was attenuated in KO mice. Recently, it was clarified that BMAL1 in PVN neurons maintains the sensitivity to GABAergic inputs from the SCN and coordinates the circadian rhythm of energy metabolism, feeding and locomotion [12,37]. The deletion of BMAL1 expression in the PVN at adulthood induces obesity and reduces circadian variation in corticosterone [12,36]. In contrast, KO mice did not exhibit obesity despite diminished circadian variation in corticosterone in this study. The HPA axis controls the homeostasis of energy metabolism and body fluid, and its role is particularly important in childhood. Therefore, it is possible that the compensatory mechanism was developed in KO mice to prevent obesity. Alternatively, the expression of *Oxt* was not affected by deletion of Bmal1 but was markedly regulated by the feeding condition. We have previously reported that nesfatin-1, a neuropeptide that is elevated by feeding, regulates circadian variation in oxytocin [20].

## 5. Conclusions

Our results indicate that BMAL1 in the PVN regulates the expression and release of AVP, thereby contributing to the maintenance of insulin secretion and glucose tolerance. In addition, the PVN delivers an efferent nerve output to the parasympathetic pre-ganglia in the pancreatic islets [38]. Future studies on the interaction of the parasympathetic signal and AVP would provide additional insights into glucose homeostasis.

## Figures and Tables

**Table 1:** Primers used in the RT-PCR experiment.

Gene	Forward	Reverse
*Avp*	5′-CATCTCTGACATGGAGCTGAGA-3′	5′-GGCAGGTAGTTCTCCTCCTG-3′
*Crh*	5′-TCTCTCTGGATCTCACCTTCCACC-3′	5′-AGCTTGCTGAGCTAACTGCTCTGC-3′
*Nucb2*	5′-GTCACAAAGTGAGGACGAGACTG-3′	5′-TGGTTCAGGTGTTCAAACTGCTTC-3′
*Oxt*	5′-TGTGCTGGACCTGGATATGCGCA-3′	5′-GGCAGGTAGTTCTCCTCCTG-3′
*Trh*	5′-TGTGACTCCTGACCTTCCA-3′	5′-GGATGCTGGCGTTTTGTG-3′
*Gapdh*	5′-GGCACAGTCAAGGCTGAGAATG-3′	5′-ATGGTGGTGAAGACGCCAGTA-3′

## Data Availability

The data used to support the findings of this study is available from the corresponding authors upon reasonable request.

## Supplementary Materials

The following are available online at https://www.mdpi.com/article/10.3390/nu13124487/s1, Figure S1: Immunostaining for BMAL1 in the supraoptic nucleus (SON), Figure S2: Corticosterone levels were measured in the serum.
