# Supplementary material for: Circadian Clock Component BMAL1 in the Paraventricular Nucleus Regulates Glucose Metabolism
Source: Nutrients. 2021 Dec 15;13(12):4487. doi: 10.3390/nu13124487 (PMC8707417; doi:10.3390/nu13124487)
Supplement: Supplementary file 1 [file nutrients-13-04487-s001.zip › nutrients-1496469-supplementary.pdf]

Supplementary material

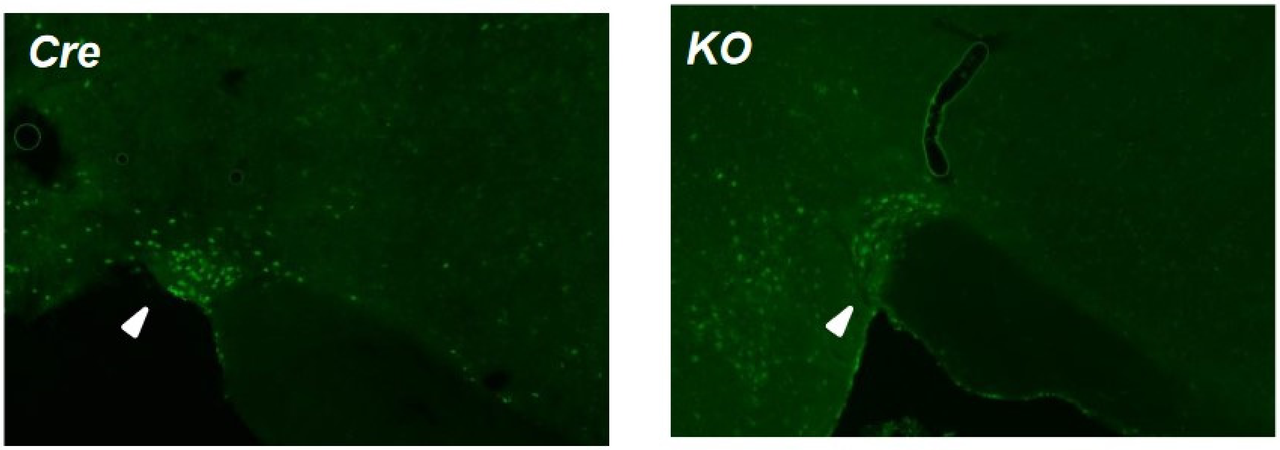

Supplementary Figure S1. Immunostaining for BMAL1 in the hypothalamic supraoptic nucleus (SON) (arrow head) of Cre and KO mice.

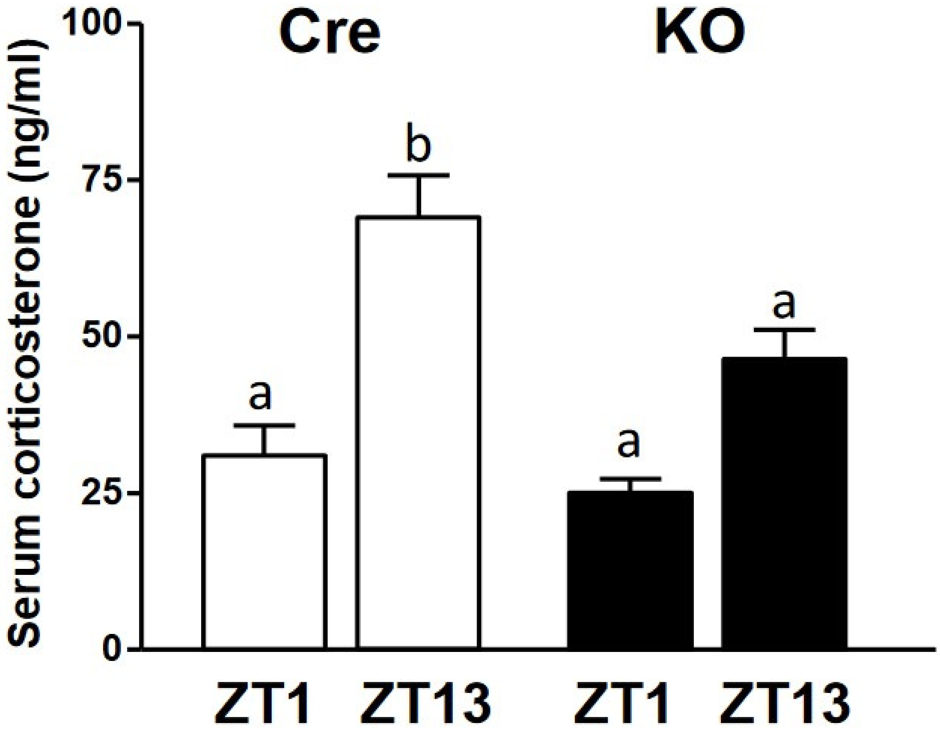

Supplementary Figure S2. Corticosterone levels were measured in the serum of at ZT1 and ZT13. Data are presented as mean ± SEM. Different letters indicate $p < 0.05$ by one- way ANOVA with Bonferroni multiple-comparison test.
